# Combined Extracts of Epimedii Folium and Ligustri Lucidi Fructus with Budesonide Attenuate Airway Remodeling in the Asthmatic Rats by Regulating Apoptosis and Autophagy

**DOI:** 10.1155/2020/2319409

**Published:** 2020-08-05

**Authors:** Zitong Ma, Xiufeng Tang, Yingying Gao, Han Wang, Ping Yu, Renhui Liu

**Affiliations:** School of Traditional Chinese Medicine, Capital Medical University, No. 10 Xitoutiao, Youanmenwai, Fengtai District, Beijing 100069, China

## Abstract

This study aimed to investigate the effects of the coadministration of budesonide (Bud) and the extracts of *Epimedii Folium* and *Ligustri Lucidi Fructus* (EEL) on regulating apoptosis and autophagy in asthmatic rats. Forty Sprague-Dawley rats were divided randomly into five groups (8 rats in each group): normal control (control), asthma model (asthma), Bud (1 mg Bud suspension in 50 ml sterile physiological saline for 30 min), EEL (100 mg/kg EEL), and group of coadministration of Bud and EEL (Bud&EEL, 100 mg/kg EEL plus Bud by nebulized inhalation for 30 min). Rats were sensitized and challenged with ovalbumin for 7 weeks and treated with corresponding drug for 4 weeks. We anesthetized all rats with 25% ethyl carbamate (4 ml/kg) and took lung tissues and BALF after final ovalbumin challenge to observe the lung histopathology and morphometry; apoptosis in BALF and lung tissue; protein expressions of Ki-67, *α*-SMA, cleaved Caspase-3, p-mTOR, and LC3; and protein and mRNA expressions of Bax, Bcl-2, Caspase-3, P53, mTOR, and Beclin-1. Results showed that Bud&EEL could alleviate airway remodeling, inhibit cell proliferation and autophagy in lung tissue, and promote apoptosis in BALF and lung tissue in ovalbumin-induced asthma rats through downregulating the protein expressions of *α*-SMA and Ki-67, the protein ratio of LC3-II/LC3-I and Bcl-2/Bax, and the protein and mRNA expressions of Bcl-2 and Beclin-1, while upregulating the protein expressions of cleaved Caspase-3 and p-mTOR, and the protein and mRNA expressions of Bax, Caspase-3, P53, and mTOR. Bud&EEL had better effects than single-use Bud on improving airway remodeling, promoting apoptosis, and regulating the expressions of autophagy- and apoptosis-related proteins. This study suggested that the effects of coadministration of EEL and Bud on regulating apoptosis and autophagy were better than those of single-use Bud treatment, and that might be the mechanism of attenuating airway remodeling, providing an alternative therapy for asthma.

## 1. Introduction

Asthma is one of the most serious chronic respiratory diseases characterized by airway inflammation, airway remodeling, and bronchial hyperresponsiveness. It affects all age brackets [1, 2], and its morbidity and mortality are increasing year by year worldwide [3]. Airway remodeling—regarded as a primary cause of refractory asthma—is characterized by epithelial shedding, goblet cell hyperplasia, basal membrane thickening, airway smooth muscle cell hyperplasia and hypertrophy, and bronchial vasculature angiogenesis [4]. It occurs in all-age asthmatic patients and can exacerbate airway inflammation and bronchial hyperresponsiveness [5]. However, researchers have not clarified the mechanism of airway remodeling and have not found the targeted treatment for it at present. Glucocorticoids (GCs), especially inhaled GCs, became the first-line treatment for asthma because of the prominent anti-inflammatory effect and rapid onset of action [6]. Inhaled GCs at conventional doses play small roles in severe asthma [7, 8], while administration at high doses or in a long term will induce a series of side effects such as growth stunting in children, GC dependent even resistance, and hypothalamic-pituitary-adrenal (HPA) axis dysfunction [9–11]. Improving therapeutic and side effects of GCs through drug combination has a great significance for the treatment of asthma.

Apoptosis and autophagy are important physiological processes to maintain cellular environmental homeostasis, participating in regulated cell death (RCD) [12]. Dysregulated RCD is related to various kinds of diseases such as cancer, autoimmune disease, and Alzheimer's disease [13]. However, RCD is especially important for lung which is exposed to the environment (pathogens and aerosolized toxins) and needs to maintain the network of epithelial-endothelial interfaces to ensure gas exchange [12]. Dysfunctions of apoptosis and autophagy in asthma have been validated in both of animal experiments and clinical studies. The activity of apoptosis in the airway biopsy of subjects with severe asthma is greater than that in normal subjects [14]. Airway inflammation is one of the most important pathological features of asthma; resolution of inflammation is regulated by production of local autacoid, apoptosis of inflammatory cells, and phagocytosis of surrounding phagocytes [15]. Reducing eosinophil apoptosis can aggravate the grade of asthma, and accumulation of eosinophils can result in airway inflammation, organ dysfunction, and airway remodeling [16]. Current and potential antiasthmatic drugs can all have effect on the number of eosinophils. Besides, in some of them, it has been verified that this effect is related to regulating eosinophils apoptosis, such as glucocorticoids, theophylline, and anti-Siglec-F [16–18]. Autophagy is a highly conserved cellular degradation process in all eukaryotes; it can degrade damaged proteins and organelle (e.g., mitochondria) to promote cellular turnover, involved in many diseases such as lung disease, heart disease, and cancer [19]. In asthma, autophagy is implicated in immune responses. It participates in regulating cell differentiation of plasmocyte, development and survival of lymphocyte, and maintaining immunological memory of B cells [20, 21]. The activity of autophagy in sputum granulocytes, peripheral blood cells, and peripheral blood eosinophils rises abnormally in severe asthmatic patients [22]. Single nucleotide polymorphism (SNP) analyses show that the variants of autophagy-related gene (ATG) allele are associated with childhood asthma and the expression of ATG5 mRNA is increased significantly in acute asthma [23]. In addition, inhibiting autophagy can improve the airway hyperresponsiveness and inflammation in ovalbumin-induced asthmatic rats [21, 24]. The interaction between apoptosis and autophagy is worth noting: autophagy-related proteins interact with apoptosis-related proteins and some proteins can have effects on both apoptosis and autophagy [25, 26]. In addition, this interaction plays a key role in airway remodeling through maintaining cellular homeostasis and mediating cell death process [27], indicating that the dysregulated apoptosis and autophagy are involved in asthmatic pathogenesis [28, 29]. This means that the regulation of apoptosis and autophagy may be one of mechanisms of improving airway remodeling in asthma.

Compounds derived from Traditional Chinese Medicine (TCM) are essential sources of seeking drugs which can improve the side and therapeutic effects of hormonotherapy. The combination of *Epimedii Folium* (EF) and *Ligustri Lucidi Fructus* (LLF) is a traditional formula and is widely used in treating asthma (also called wheeze syndrome in TCM), achieving excellent curative effect. The extracts of EF and LLF (EEL) are mainly icariin and oleanolic acid. EF and LLF are used widely in the treatment of osteoporosis, cancer, sexual dysfunction, and asthma and have the functions of increasing bone mineral density and adjusting sex hormone, in addition to their antioxidative and anti-inflammatory effects [30–32]. There are also many studies which show the effects of EF and LLF on apoptosis and autophagy. Both of EF and LLF can regulate apoptosis by affecting Caspase-3 [33]. Icariin, an active ingredient of EF, suppresses oxygen-glucose deprivation and reperfusion-induced apoptosis and autophagy in PC12 cells [34]. Oleanolic acid, an active ingredient of LLF, has anticancer effects by regulating intracellular environment: it can promote apoptosis of HL60 cells through death-receptor pathway [35] and induce autophagy of bladder cancer cells through AMPK-mTOR-ULK1 signaling pathway [36].

In previous studies, we found that the coadministration of EEL and GCs (dexamethasone and budesonide) had better effects on airway remodeling and inflammation by reducing the levels of interleukin-4 (IL-4), IL-5, and IgE in serum and inhibiting the TGF-*β*1/Smad pathway in ovalbumin-induced asthmatic rats compared with single-use GC treatment [37, 38]; EEL can increase the sensitivity of asthmatic rats to budesonide by promoting lymphocyte apoptosis and balancing GR/HSP90 [39]. We speculate that the coadministration of EEL and GC could act synergistically to restore the dysregulated apoptosis and autophagy involved in asthmatic pathogenesis based on the interaction between apoptosis and autophagy. In the present study, we selected ovalbumin-induced asthma rats to explore and compare the effects of EEL and budesonide separately or combinedly in apoptosis and autophagy.

## 2. Methods

### 2.1. Preparation of Active Ingredients

*Epimedii Folium* (EF, dried leaf of *Epimedium brevicornum* Maxim) and *Ligustri Lucidi Fructus* (LLF, dried mature seed of *Ligustrum lucidum* Ait.) were purchased from Beijing Tongrentang pharmaceutical Co. Ltd., China. They were authenticated by an expert herbalist, Shiyuan Jin, Honorary Professor, School of TCM, Capital Medical University. Voucher specimens were deposited at the TCM Endocrine and Metabolic Disease Laboratory of TCM School of Capital Medical University, China. Preparation of active ingredients of EF and LLF, including flavonoids and iridoids, was performed according to the methods described before [40]. And the process of extracting combined active ingredients has been protected by the Chinese patent 20140037992.5. The active ingredients of EF and LLF were mixed at a ratio of 2 to 3, equivalent to the raw herbal ratio of 4 to 3 according to clinical practice. Before application, the combined active fractions were dissolved with distilled water at 10 mg/ml.

### 2.2. Animals

40 male Sprague-Dawley rats, weighing 120 to 130 g, were purchased from Vital River Laboratory Animal Technology Co. Ltd. (Beijing, China). he experiment complied with the Animal Management Rule of Ministry of Public THealth, China, and the experimental protocol was approved by Animal Care Committee of Capital Medical University, Beijing, China. All the animals were cared for in Experimental Animal Center of Capital Medical University. During the whole experiment, the animals were housed in stainless cages (three rats per cage) at conventional controlled conditions (temperature of 23 ± 2°C, relative humidity of 50 ± 10%, and 12-hour light-dark cycle). They were allowed free access to the standard laboratory food and tap water.

### 2.3. Experimental Protocol

**Scheme 1:**
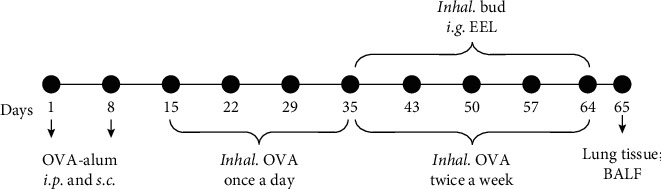
The experimental protocol. Following the sensitization and challenges of ovalbumin, rats were treated with Bud, EEL, or distilled water on days 35–64 and then sacrificed with acquisition of lung tissue and BALF on day 65. OVA-alum: ovalbumin dissolved in aluminum hydroxide adjuvant; *i.p.*: intraperitoneal injection; *s.c.*: subcutaneous injection; *Inhal*.: nebulized inhalation; *i.g.*: gastric perfusion.

After acclimatization for 7 days, the rats were randomly assigned to 5 groups (*n* = 8 per group): normal control group (control), asthma model group (asthma), budesonide group (Bud), group of extracts of EF and LLF (EEL), and group of coadministration of Bud and EEL (Bud&EEL). The experimental protocols were performed according to our previous study [39], as explained in Scheme 1. All rats except rats in control group were sensitized with 1 mg ovalbumin (Grade II, Sigma-Aldrich, St. Louis, MO, USA) and 100 *μ*g aluminum hydroxide (Sigma) in 1 ml sterile physiological saline by intraperitoneal injection (*i.p.* 0.5 ml) and subcutaneous injection (*s.c.*; 5 spots: hind feet on both sides, groins on both sides, and back; every spot was injected with 0.1 ml.) on the 1st and 8th days and challenged with 1 mg ovalbumin in 100 ml sterile physiological saline by nebulized inhalation with a flow of 0.8 ml/min for 30 min for 7 weeks. Rats in control group were treated with sterile physiological saline. On the 35th to 64th days, rats were administrated with corresponding drug once a day. Control and asthma groups were treated with isometric distilled water; Bud group was treated with Bud (1 mg Bud suspension in 50 ml sterile physiological saline, AstraZeneca Pty Ltd., New South Wales, Australia) by nebulized inhalation with a flow of 1.6 ml/min for 30 min; EEL group was treated with EEL (100 mg/kg body weight) by gavage; Bud&EEL group was treated with both of Bud and EEL. On the 65th day, we anesthetized all rats with 25% ethyl carbamate (4 ml/kg body weight, *i.p.*), opened rat's chest, and took out lung tissue along with trachea.

### 2.4. Lung Histopathology and Morphometry

The middle lobe of the left lung was cut off and fixed by 4% paraformaldehyde, for preparation to be embedded by paraffin, and then routinely processed. Lung tissue sections were stained with hematoxylin and eosin (H&E), periodic acid–Schiff (PAS), and Masson's trichrome and then measured with the Nikon ECLIPSE 80i biomicroscope and NIS-Elements BR 3.2 image analysis system (Nikon, Japanese) according to our previous study [38].

We surveyed the perimeter of basement membrane (Pbm), total area of bronchus (Wat1), area of lumen (Wat2), area of outer margin of the smooth muscle (Wam1), and area of medial smooth muscle (Wam2) in H&E-stained sections; then calculated the standardized thickness of airway wall (Wat, Wat = (Wat1 − Wat2)/Pbm) and the standardized thickness of airway smooth muscle (Wam, Wam = (Wam1 − Wam2)/Pbm); observed the goblet cells and Pbm in PAS-stained sections; then calculated the standardized number (number/Pbm) and area (area/Pbm) of PAS-positive goblet cells; determined collagen fiber area (stained in blue) and Pbm in Masson's trichrome-stained lung sections; and then calculated the mean score of the fibrotic area divided by Pbm in each rat.

### 2.5. TdT-Mediated dUTP Nick-End Labeling (TUNEL) Assay for Apoptosis in BALF and Lung Tissue

The cell-debris pellets of bronchoalveolar lavage fluid (BALF) samples and lung tissue (paraffin sections) were prepared to determine apoptosis activity using the TUNEL assay (Beyotime Co., Ltd., Shanghai, China) according to manufacturer's instructions.

### 2.6. Immunofluorescence Analysis (IF)

We mainly used paraffin-embedded lung sections for immunofluorescent staining. The paraffin sections were dewaxed with xylene, ethanol, and double distilled water, antigen-retrieved with citrate buffer using microwave oven, and blocked with 10% normal goat serum for 2 hours. Then, they were incubated with primary antibodies against alpha smooth muscle actin (*α*-SMA), Ki-67, Bcl-2, Bax, Caspase-3, P53, mTOR, and Beclin-1, respectively, overnight at 4°C. On the second day, the sections were incubated with relevant fluorescent secondary antibodies (Keygen Biotech Co., Ltd., Jiangsu, China) at 37°C for 2 h and mounted with antifade mounting medium containing DAPI (Solarbio Science & Technology Co., Ltd., Beijing, China). At least 3 views with airway were analyzed per rat in fluorescence microscope. All measurements were performed with the Nikon ECLIPSE 80i biomicroscope and NIS-Elements BR 3.2 image analysis system (Nikon, Japanese). The description and concentration of primary antibodies used in IF analysis are listed in Table 1.

### 2.7. Western Blot Analysis (WB)

The total protein was extracted from 60 mg lung tissue using RIPA lysis buffer. Concentration of the protein was quantified by quantitative bromochloroacetic acid (BCA) protein kit (Beijing Biosynthesis Biotechnology Co., Ltd., Beijing, China). The protein was mixed with 4 × SDS-PAGE loading buffer (dilution rate 3 : 1) and boiled at 100°C for 10 min to make it denatured. Equal amounts of protein (30 *μ*g *per* lane) were separated by sodium dodecyl sulfate polyacrylamide gel electrophoresis (SDS-PAGE) and transferred into 0.45 *μ*m polyvinylidene fluoride (PVDF) membranes. After blocking with 5% nonfat-dried milk at room temperature for 2 h, membranes were incubated with primary antibodies overnight at 4°C. Membranes were detected with relevant horseradish peroxidase-labeled secondary antibodies (Zhong Shan Golden Bridge Biotechnology Co., Ltd., Beijing, China; 1 : 20000) for 1 h. The protein band was visualized by an electrochemiluminescent (ECL) reagent and exposed to X-film. GAPDH or *β*-actin was used for normalization. The mean density of each protein band was measured by ImageJ software (National Institutes of Health, USA). The description and concentration of primary antibodies used in WB analysis are listed in Table 2.

### 2.8. Quantitative Real-Time PCR Analysis (qPCR)

Total RNA was isolated from the lung tissue with the RNAprep pure Tissue Kit (TianGen Co., Ltd., Beijing, China) according to the manufacturer's recommendations. Total RNA (2 *μ*g) was reverse-transcribed using the FastKing RT Kit (with gDNase) (TianGen Co., Ltd., Beijing, China) to generate complementary DNA (cDNA). SuperReal PreMix Plus (SYBR-green) and Bio-Rad PCR cycler (CFX96 Real-Time System) were used for real-time quantitative analysis. The PCR program was performed for 40 cycles with each cycle consisting of 12 s of denaturation at 95°C, 1 min of annealing at 60°C, and 10 s of extension at 60°C. The mRNA expression was quantified as previously described [41]. The primers used in the qPCR analysis are presented in Table 3.

### 2.9. Statistical Analysis

Results of all measurements were presented as means ± standard deviation (SD). The statistical differences (*P* < 0.05) among experimental groups were evaluated using SPSS 21.0 software (SPSS Inc., Chicago, USA) by one-way analysis of variance (ANOVA). The least significant difference (LSD) test when the variances were equal or Tamhane's T2 test when the variances were not equal was used for comparisons between individual groups and to determine which means differed statistically significantly (*P* < 0.05).

## 3. Results

### 3.1. Effects of Bud and EEL on Airway Remodeling

**Figure 1:**
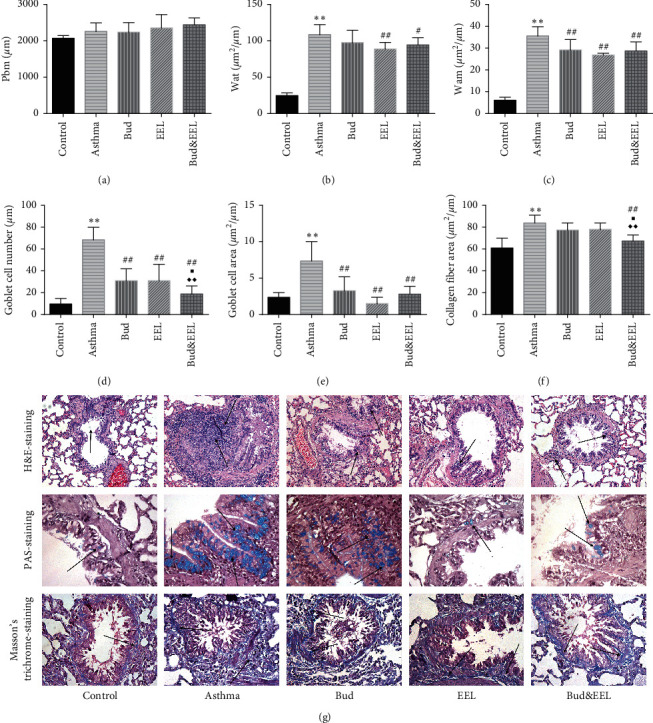
Effects of bud and EEL on airway remodeling. The inflammatory cell infiltration and airway remodeling were observed by (a) the perimeter of basement membrane (Pbm), (b) the standardized thickness of airway wall (Wat), and (c) the standardized thickness of airway smooth muscle (Wam) in H&E-stained lung sections. The goblet cell hyperplasia was assessed by (d) the number of goblet cell and (e) the area of goblet cell in PAS-stained lung sections. (f) The area of collagen fiber in Masson's trichrome-stained lung sections. (g) Representative photomicrographs of H&E-stained, PAS-stained, and Masson's trichrome-stained lung sections from each group (×400). Data are represented as mean ± SD; *n* = 6. ^*∗∗*^*P* < 0.01 compared with control group; ^#^*P* < 0.05 and ^##^*P* < 0.01 compared with asthma group; ^■^*P* < 0.05 compared with Bud group; ^◆◆^*P* < 0.01 compared with EEL group.

We observed the Pbm, Wat, and Wam to assess the features of inflammatory cell infiltration and airway remodeling in H&E-stained lung sections. As shown in Figure 1(g), no inflammation, mucosal edema, or epithelial lesions were observed in the control group. However, severe inflammation, mucosal edema, epithelial lesions, and inflammatory cell infiltration including lymphocyte and eosinophil were observed in asthma model group which were attenuated by administration of Bud, EEL, or Bud&EEL. Wat and Wam in the asthma group were greater than those in the control group (Figures 1(b) and 1(c); all *P* < 0.01). Compared with asthma group, Wat was remarkably decreased in EEL and Bud&EEL groups (*P* < 0.05 or 0.01); Wam was significantly reduced in all three treatments (all *P* < 0.01). There were no significant differences in Pbm in all groups (Figure 1(a)).

Goblet cell hyperplasia and collagen deposition are implicated in mucus hypersecretion and collagen hyperplasia, which are the major airway remodeling features in asthma and can induce airflow limitation [42, 43]. We observed goblet cell hyperplasia in the airway epithelium of asthmatic rats by the PAS staining, but not in control rats (Figure 1(g)). The number and area of goblet cell were increased significantly in asthma group vs. control group (Figures 1(d) and 1(e); all *P* < 0.01) and were decreased after drug administration of each group (all *P* < 0.01), illuminating that the treatment of Bud and EEL could restrain the goblet cell hyperplasia in ovalbumin-induced asthmatic rats either separately or in combination. The inhibitory effect of Bud&EEL on goblet cell hyperplasia was better than that of Bud or EEL (*P* < 0.05 or 0.01).

Collagen deposition in the rat airway wall was observed by Masson's trichrome staining to assess the level of collagenous hyperplasia. The area of collagen fiber was significantly greater in asthma group than that in control group (Figures 1(f) and 1(g); *P* < 0.01). After treatment, Bud&EEL notably reduced the area of collagen fiber compared with asthma or Bud group (*P* < 0.05 or 0.01). These findings indicate that the combination of Bud and EEL has a better effect on alleviating airway remodeling.

### 3.2. Effects of Bud and EEL on Airway Smooth Muscle Cells Hyperplasia

**Figure 2:**
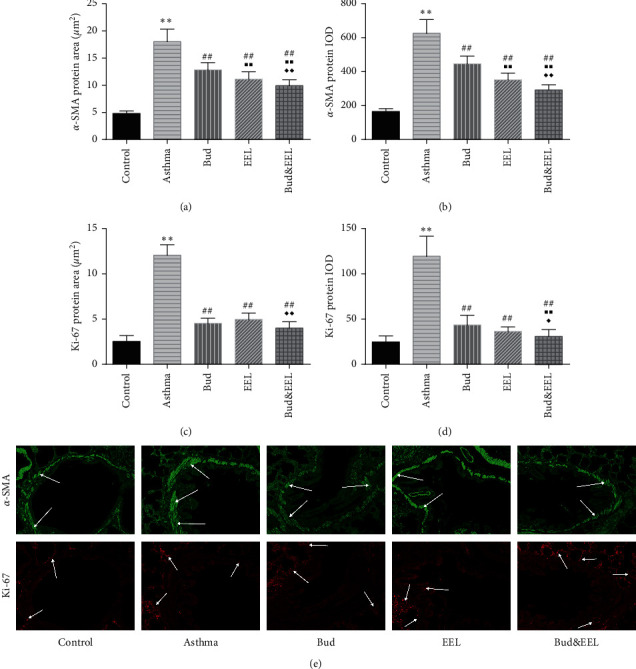
Effects of bud and EEL on cell proliferation in lung tissue. The positive (a) area and (b) IOD of *α*-SMA. The positive (c) area and (d) IOD of Ki-67. (e) Representative immunofluorescence images of *α*-SMA and Ki-67 (×200). Data are represented as mean ± SD; *n* = 6. ^*∗∗*^*P* < 0.01 compared with control group; ^##^*P* < 0.01 compared with asthma group; ^■■^*P* < 0.01 compared with Bud group; ^◆^*P* < 0.05 and ^◆◆^*P* < 0.01 compared with EEL group.

High expression of *α*-SMA protein in airway smooth muscle cells is closely associated with airway remodeling and pulmonary fibrosis. Ki-67, regarded as a proliferative marker, plays a key role in epithelial cell proliferation and differentiation [44]. We examined *α*-SMA and Ki-67 by IF to investigate the effect of the coadministration of Bud and EEL on airway smooth muscle cell hyperplasia in ovalbumin-induced asthmatic rat airways (Figure 2(e)).

Positive area and integral optical density (IOD) of *α*-SMA and Ki-67 in asthma group were significantly greater than those in control group (Figures 2(a) and 2(d); all *P* < 0.01). After administration, all three treatments notably inhibited the protein expressions of *α*-SMA and Ki-67 vs. asthma group (all *P* < 0.01). Bud&EEL significantly downregulated the two proteins compared with Bud or EEL (*P* < 0.05 or 0.01). These data suggest that the effect of combination of Bud and EEL on reducing the airway smooth muscle cells hyperplasia was better than Bud or EEL in asthmatic rats.

### 3.3. Effects of Bud and EEL on Apoptosis in BALF and Lung Tissue

**Figure 3:**
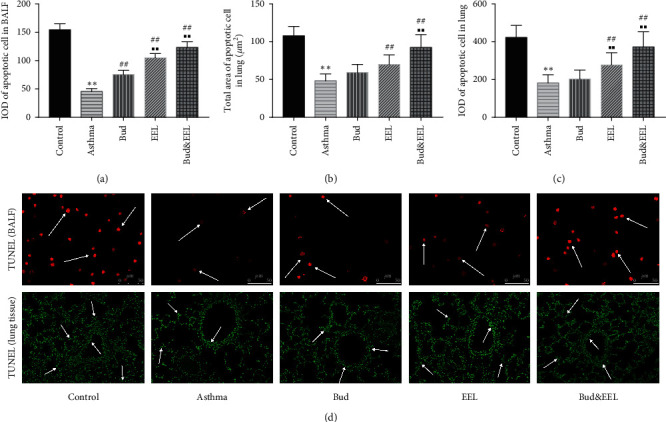
Effects of bud and EEL on apoptosis in BALF and lung tissue. (a) The IOD of fluorescein-dUTP in BALF cells. The positive (b) area and (c) IOD of fluorescein-dUTP in lung tissue. (d) Representative immunofluorescence images of TUNEL in BALF (×400) and in lung tissue (×200). Red: apoptosis in BALF; green: apoptosis in lung tissue. Data are represented as mean ± SD; *n* = 6. ^*∗∗*^*P* < 0.01 compared with control group; ^##^*P* < 0.01 compared with asthma group; ^■■^*P* < 0.01 compared with Bud group.

The high-level apoptosis of epithelial cells and low-level apoptosis of immune cells are the important pathogenesis of asthma [12]. In the present study, we used TUNEL assay to detect apoptosis in BALF and lung tissue. Ovalbumin depressed lymphocyte apoptosis in BALF (Figure 3(a); *P* < 0.01), which was determined by IOD of fluorescence labeling. Bud, EEL, and Bud&EEL significantly induced apoptosis in BALF compared with asthma group (all *P* < 0.01). In addition, there was a significant difference in apoptosis of BALF between Bud group and Bud&EEL group (*P* < 0.01).

Similarly, apoptosis activity in lung tissue was inhibited in asthma group, and the inducing apoptosis activity of EEL and Bud&EEL was found, which was determined by positive area and IOD of fluorescence labeling (Figures 3(b) and 3(c); *P* < 0.01). The effect of Bud&EEL on inducing apoptosis in lung tissue was better than that of Bud (*P* < 0.01). We can infer from these data that apoptosis in BALF and lung tissue is decreased in ovalbumin-induced asthmatic rats, which can be promoted by the combination of Bud and EEL, suggesting that this positive effect might be associated with improving airway remodeling.

### 3.4. Effects of Bud and EEL on Bcl-2 and Bax

**Figure 4:**
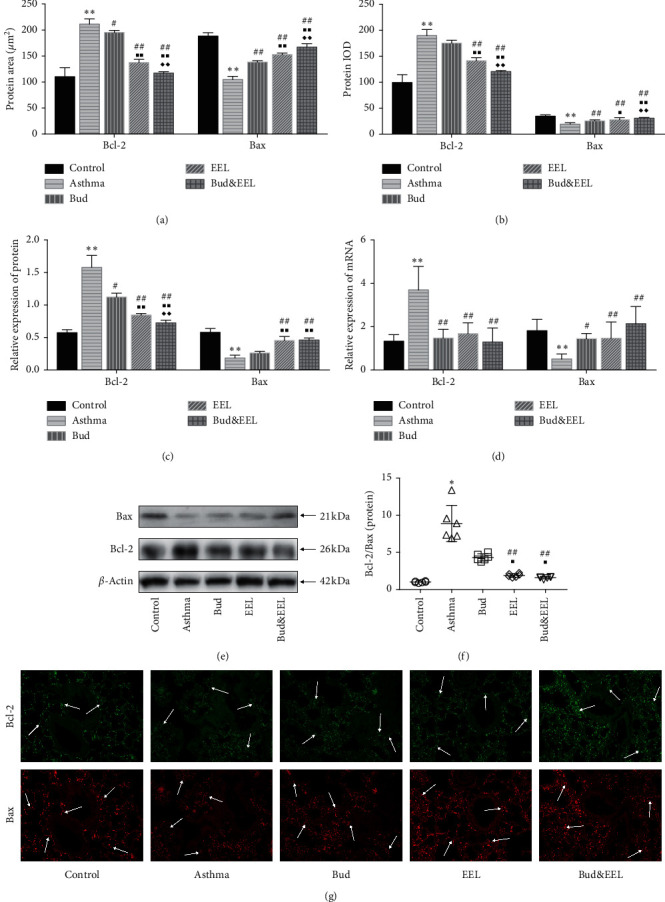
Effects of bud and EEL on Bcl-2 and Bax. The protein area (a) and IOD (b) of Bcl-2 and Bax were measured by immunofluorescence staining. (c) Summarized data of mean density of Bcl-2 and Bax protein expression in lung tissues measured by WB, normalized to *β*-actin. (d) The mRNA expressions of Bcl-2 and Bax were measured by qPCR analysis with *β*-actin as an internal control. (e) Representative WB photographs of Bcl-2 and Bax were viewed, and *β*-actin was used for normalization. (f) The ratio of Bcl-2 to Bax was calculated in the level of relative protein measured by WB. (g) Representative immunofluorescence images of Bcl-2 and Bax (×200). Data are represented as mean ± SD; *n* = 6. ^*∗*^*P* < 0.05 and ^*∗∗*^*P* < 0.01 compared with control group; ^#^*P* < 0.05 and ^##^*P* < 0.01 compared with asthma group; ^■^*P* < 0.05 and ^■■^*P* < 0.01 compared with bud group; ^◆◆^*P* < 0.01 compared with EEL group.

According to the different biological functions and structural domain, the Bcl-2 family, the first discovered cell death regulator, can separate into two types: apoptosis-promoting proteins (Bax, Bak) and apoptosis-inhibiting proteins (Bcl-2, Bcl-xL) [45]. In addition, recent studies have indicated that Bcl-2 can not only inhibit autophagy by binding Beclin-1 [46] but also resist glucocorticoid-induced apoptosis [47]. As shown in Figures 4(a) and 4(b), positive area and IOD of Bcl-2 protein detected by IF were increased significantly whereas those of Bax protein were decreased in asthma group vs. control group (all *P* < 0.01). Compared with asthma group, the positive area of Bcl-2 was remarkably decreased, whereas the positive area and IOD of Bax were increased in all three treatment groups (*P* < 0.05 or 0.01); EEL and Bud&EEL reduced significantly the IOD of Bcl-2 (all *P* < 0.01). Bud&EEL notably downregulated Bcl-2 protein and upregulated Bax protein compared with Bud or EEL (all *P* < 0.01).

To further investigate the effects of Bud and EEL on Bcl-2 and Bax, we confirmed the protein of Bcl-2 and Bax by WB analysis (Figures 4(c) and 4(e)) and detected the mRNA expressions of Bcl-2 and Bax by qPCR (Figure 4(d)). Bcl-2 protein and mRNA were significantly upregulated, while Bax protein and mRNA were downregulated in asthma group vs. control group (all *P* < 0.01). After administration, all three treatments significantly inhibited the protein and mRNA expression of Bcl-2 and promoted mRNA expression of Bax (*P* < 0.05 or 0.01); EEL and Bud&EEL upregulated Bax protein (all *P* < 0.01). We observed that Bud&EEL significantly decreased Bcl-2 protein and increased Bax protein compared with Bud group (all *P* < 0.01).

The imbalance between the Bcl-2 family proteins, which has been verified in asthmatic rat lung tissues, can impact mitochondria apoptosis pathway [48]. We calculated the protein ratio of Bcl-2 to Bax measured by WB to assess the effect of Bud and ELL on balancing the protein expression of Bcl-2 family. The protein ratio of Bcl-2/Bax was notably increased in the asthma group vs. the control group (Figure 4(f); *P* < 0.05). Compared with the asthma or Bud group, Bcl-2/Bax was significantly decreased in EEL and Bud&EEL groups (*P* < 0.05 or 0.01). These results indicate that coadministration of Bud and EEL can regulate Bcl-2 and Bax and balance Bcl-2/Bax in asthmatic rats. Further, EEL has synergistic effect on regulating Bcl-2 and Bax with Bud.

### 3.5. Effects of Bud and EEL on Caspase-3 and Cleaved Caspase-3

Caspase-3 is an executor caspase, playing an essential role in the final phase of apoptosis [49]. Cleaving proCaspase-3 to generate the active Caspase-3 heterodimer (cleaved Caspase- 3) is a necessary step in activating apoptosis [50]. In addition, Caspase-3, as a regulator of autophagy, can inhibit autophagy through cleaving autophagy-related gene (ATG), such as ATG3 and ATG4 [51, 52].

**Figure 5:**
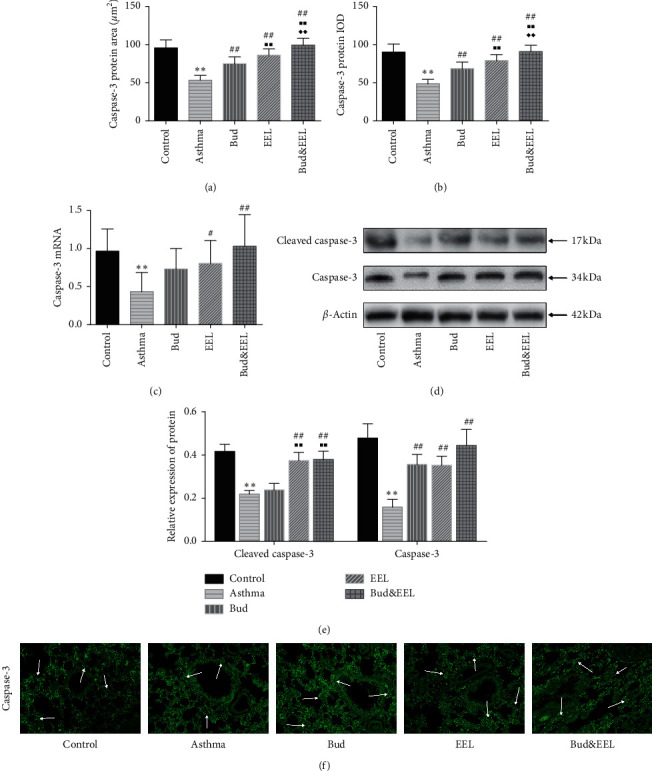
Effects of bud and EEL on Caspase-3 and cleaved Caspase-3. The protein area (a) and IOD (b) of Caspase-3 were measured by immunofluorescence staining. (c) The mRNA expressions of Caspase-3 were measured by qPCR analysis with *β*-actin as an internal control. (d) Representative WB photographs of Caspase-3 and cleaved Caspase-3 were viewed, and *β*-actin was used for normalization. (e) Summarized data of mean density of cleaved Caspase-3 and Caspase-3 protein expression in lung tissues measured by WB, normalized to *β*-actin. (f) Representative immunofluorescence images of Caspase-3 (×200). Data are represented as mean ± SD; *n* = 6. ^*∗∗*^*P* < 0.01 compared with control group; ^#^*P* < 0.05 and ^##^*P* < 0.01 compared with asthma group; ^■■^*P* < 0.01 compared with Bud group; ^◆◆^*P* < 0.01 compared with EEL group.

We observed that Caspase-3 protein expression detected by IF was significantly reduced in the asthma group vs. the control group (Figures 5(a) and 5(b); all *P* < 0.01) and was increased after drug administration of each group (all *P* < 0.01); Caspase-3 mRNA expression by qPCR was significantly decreased in asthma group vs. control group (Figure 5(c); *P* < 0.01) and was upregulated after the treatment of EEL and Bud&EEL (*P* < 0.05 or 0.01).

To further investigate the effects of Bud and EEL on Caspase-3 protein, we used WB analysis to detect Caspase-3 and cleaved Caspase-3 (Figures 5(d) and 5(e)). We found that Caspase-3 and cleaved Caspase-3 were reduced significantly in the asthma group vs. the control group (all *P* < 0.01). EEL and Bud&EEL significantly promoted cleaved Caspase-3 protein, and all three treatments remarkably promoted Caspase-3 protein compared with asthma group (all *P* < 0.01). These results suggest that the coadministration of Bud and EEL has a better effect on activating Caspase-3 than single-use Bud treatment.

### 3.6. Effects of Bud and EEL on LC3 and Beclin-1

The macroautophagy, a primary form of autophagy, participates in asthmatic pathogenesis. LC3 and Beclin-1 proteins can promote autophagy by taking part in the formation of autophagosome which is an important procedure of macroautophagy [53, 54]. To investigate the effects of Bud and EEL on autophagy, we detected Beclin-1 and LC3.

**Figure 6:**
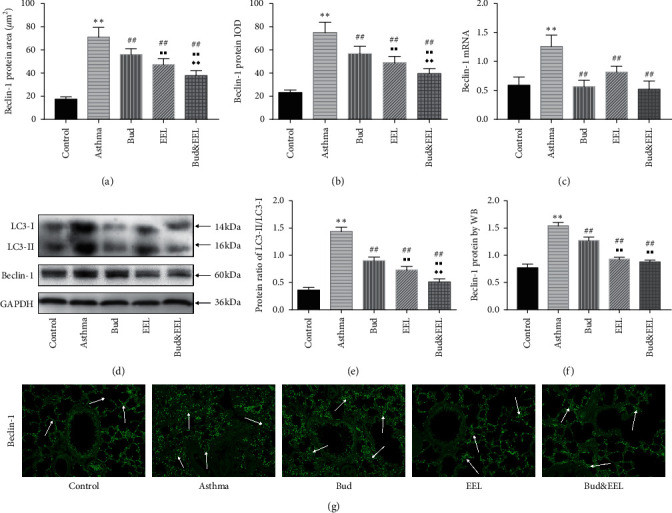
Effects of Bud and EEL on LC3 and Beclin-1. The protein area (a) and IOD (b) of Beclin-1 were measured by immunofluorescence staining. (c) The mRNA expressions of Beclin-1 were measured by qPCR analysis with *β*-actin as an internal control. (d) Representative WB photographs of LC3 and Beclin-1 were viewed, and GAPDH was used for normalization. Summarized data of mean density of protein ratio of LC3-II/LC3-I (e) and Beclin-1 protein (f) in lung tissues measured by WB, normalized to GAPDH. (g) Representative immunofluorescence images of Beclin-1 (×200). Data are represented as mean ± SD; *n* = 6. ^*∗∗*^*P* < 0.01 compared with control group; ^##^*P* < 0.01 compared with asthma group; ^■■^*P* < 0.01 compared with Bud group; ^◆◆^*P* < 0.01 compared with EEL group.

Beclin-1 protein detected by both IF and WB, and Beclin-1 mRNA detected by qPCR were increased significantly in asthma group vs. control group (Figures 6(a), 6(d), and 6(f); all *P* < 0.01), and were decreased after drug administration of each group (all *P* < 0.01). Compared with Bud group, Beclin-1 protein was downregulated remarkably in EEL and Bud&EEL groups (all *P* < 0.01).

We measured the protein expressions of LC3 by WB and calculated the ratio of LC3-II to LC3-I. LC3-II/LC3-I was upregulated remarkably in the asthma group vs. the control group (Figures 6(d) and 6(e); *P* < 0.01). After administration, all three treatments notably reduced LC3-II/LC3-I compared with asthma group (all *P* < 0.01), indicating that the activity of autophagy in asthmatic rat lung tissue was promoted, which could be resisted with the treatment of Bud and EEL. In addition, there was significant decrease in EEL and Bud&EEL groups compared with Bud group (all *P* < 0.01). These results suggested that the inhibitory effect of Bud&EEL on autophagy was better than that of single-Bud treatment in ovalbumin-induced rats, indicating that might be a potential mechanism of synergistic effect of EEL with Bud on airway remodeling.

### 3.7. Effects of Bud and EEL on P53, mTOR, and p-mTOR

The relationship between apoptosis and autophagy is very complicated, because some proteins can influence the activity of both apoptosis and autophagy such as P53 and mTOR. P53 can promote the expression of many proapoptotic proteins such as Bax, Apaf-1, and Caspase-6 and ultimately induce apoptosis. What is more, nuclear p53 promotes autophagy, whereas cytoplasmic p53 prevents it. Both functions work through impacting AMPK-TSC2-mTOR pathway. mTOR inhibits autophagy and apoptosis and affects cell cycle regulation, proliferation, and differentiation [55, 56]. mTORC1 and mTORC2 are two complexes formed by mTOR. Phospho-mTOR (Ser2448) is mainly regulated by mTORC1 which can downregulate autophagy.

**Figure 7:**
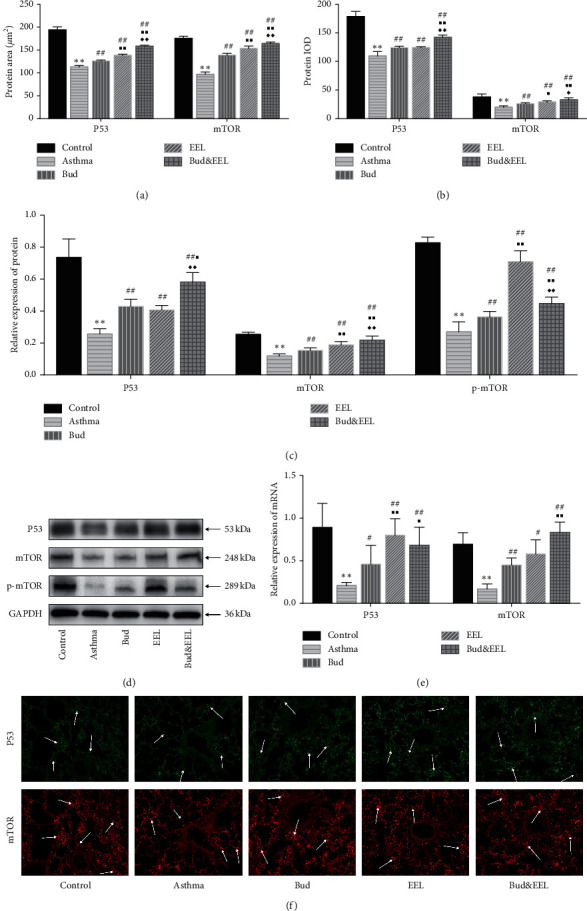
Effects of Bud and EEL on P53, mTOR, and p-mTOR. The protein area (a) and IOD (b) of P53 and mTOR were measured by immunofluorescence staining. (c) Summarized data of mean density of P53, mTOR, and p-mTOR protein expression in lung tissues measured by WB, normalized to GAPDH. (d) Representative WB photographs of P53, mTOR, and p-mTOR were viewed, and GAPDH was used for normalization. (e) The mRNA expressions of P53 and mTOR were measured by qPCR analysis with *β*-actin as an internal control. (f) Representative immunofluorescence images of P53 and mTOR (×200). Data are represented as mean ± SD; *n* = 6. ^*∗∗*^*P* < 0.01 compared with control group; ^#^*P* < 0.05 and ^##^*P* < 0.01 compared with asthma group; ^■^*P* < 0.05 and ^■■^*P* < 0.01 compared with Bud group; ^◆^*P* < 0.05 and ^◆◆^*P* < 0.01 compared with EEL group.

We found that the protein and mRNA of P53 and mTOR, detected by IF and qPCR, were significantly reduced in the asthma group vs. the control group (Figures 7(a), 7(b), and 7(e); all *P* < 0.01). Compared with asthma group, P53 and mTOR in both protein and mRNA level were upregulated significantly in all three treatment groups (*P* < 0.05or 0.01). The effect of Bud&EEL on upregulating P53 and mTOR was better than that of Bud (*P* < 0.05 or 0.01).

To further confirm the effects of the Bud and EEL on P53 and mTOR, we detected the protein expressions of P53, mTOR, and p-mTOR in rat lung tissue by WB analysis (Figures 7(c) and 7(d)). The protein expressions of P53, mTOR, and p-mTOR were reduced remarkably in asthma group vs. control group (all *P* < 0.01) and were increased after drug administration of each group (all *P* < 0.01). Bud&EEL significantly increased the protein expression of P53, mTOR, and p-mTOR compared with Bud (*P* < 0.05 or 0.01). These results suggest that EEL could activate P53 and mTOR in asthmatic rat lung tissue, which might be associated with the synergistic effect of EEL with Bud on airway remodeling.

## 4. Discussion

Airway remodeling is an irreversible change in airway structure and function induced by chronic impairment, relating to severity of asthma [57, 58]. At present, asthmatic treatments (including GCs and long-acting *β*2-agonist and bronchodilators) have little influence on airway remodeling, the primary cause of refractory asthma [59]. In our study, Bud&EEL relieved the incrassation of the airway wall and smooth muscle, goblet cell hyperplasia, and collagen deposition in ovalbumin-induced asthma rats. In addition, Bud&EEL significantly reduced goblet cell number and alleviated collagen deposition, compared with Bud. These findings indicate that the combination of EEL with Bud might have a better effect on the pathological changes of airway remodeling, especially the collagen deposition and goblet cell hyperplasia.

Airway smooth muscle cells are not only the secondary effector cells but also the incipient inductor cells in airway remodeling [4]. *α*-SMA reflects the scale and shrink capability of smooth muscle cells which induce tracheal stenosis and contraction [60], highly expressed in myofibroblasts [61] and negatively correlated with lung function in vitro asthmatic experiments [62]. Ki-67 is regarded as a crucial marker of cell proliferation which is highly expressed in cycling cells but lowly expressed in resting Go cells [63, 64]. In order to further confirm the effect of Bud&EEL on airway remodeling, we investigated hyperplasia of airway smooth muscle cells by detecting *α*-SMA and Ki-67 in lung tissue. We found that *α*-SMA and Ki-67 were remarkably increased in asthma rats, consistent with the pathological changes of airway remodeling. EEL, Bud, and Bud&EEL significantly reduced *α*-SMA and Ki-67. Bud&EEL had the priority in downregulating *α*-SMA and Ki-67 compared to Bud, suggesting that the coadministration of EEL and Bud has synergistic effects on airway smooth muscle cell hyperplasia in ovalbumin-induced asthma rats.

As an apoptosis inducer, GCs' antiremodeling effect is related to regulating apoptosis. We found that Bud&EEL reduced apoptosis in BALF and lung tissue, and this influence was more effective than that of single-Bud treatment. In order to investigate the molecular mechanism of that, we detected Bcl-2, Bax, Caspase-3, and P53 in protein and mRNA. In the Bcl-2 protein family, Bcl-2 (apoptosis-inhibiting protein) and Bax (apoptosis-promoting protein) play important roles in mitochondrial apoptosis pathway by regulating the mitochondrial release of cytochrome c [65, 66]. In the Caspases family, Caspase-3 is a key protease in mitochondria-dependent and -independent apoptosis pathways, executing final phase of apoptosis [67, 68]. Remarkably, Bcl-2 and Bax are upstream regulators of Caspase-3 in intrinsic apoptosis pathway [69, 70]. P53, playing an essential role in mediating apoptosis, cell cycle arrest, and DNA repair, can improve the activity of apoptosis by upregulating proapoptosis genes [71]. For example, P53 can bind to Bcl-2, promoting the release of Bax and enhancing the activity of Caspase-3 [72, 73]. We observed that ovalbumin depressed apoptosis activity and proapoptosis factors including Bax, Caspase-3, and P53 increased Bcl-2 and Bcl-2/Bax. Bud, EEL, and Bud&EEL upregulated apoptosis in BALF, Bax, and P53 in protein and mRNA levels but reduced Bcl-2. In addition, EEL and Bud&EEL could upregulate apoptosis in lung tissue and the expressions of cleaved Caspase-3 and Caspase-3 but decrease Bcl-2/Bax. Moreover, Bud&EEL had better effects on regulating apoptosis, Bcl-2, Bax, P53, and Caspase-3 than Bud. The data suggest that promoting apoptosis might be a possible mechanism by which the coadministration of Bud and EEL improves airway remodeling in asthma.

Autophagy plays a pivotal role in maintaining cellular homeostasis through elimination of misfolded proteins, protein aggregates, and damaged organelles in physiology situations [74]. It can be divided into three categories: microautophagy, chaperone-mediated autophagy (CMA), and macroautophagy. Macroautophagy (hereafter referred to as “autophagy”) is implicated in airway inflammation and airway remodeling in asthma [20]. In mediating airway remodeling process, autophagy in airway epithelial cells can induce epithelial-mesenchymal transition in airways, leading to aggravation of airway remodeling and pulmonary fibrosis [75]. In order to observe the autophagic activity in asthmatic rat and the effects of Bud and EEL on that, we detected the expressions of Beclin-1, LC3, and mTOR which play crucial roles in regulating autophagy. Under conditions of nutrient deprivation or cellular stress, AMPK activates phosphorylation of ULK and Beclin-1, triggering autophagy [76]; mTORC1 is a complex of mTOR which can catalyze the inactivating phosphorylation of ULK1, suppressing autophagy [77]. Beclin-1 can activate ATG and promote the conversion of cytoplasmic soluble LC3-I to membrane combined LC3-II to accelerate formation of autophagosome, inducing autophagy. Therefore, the protein ratio of LC3-II to LC3-I is regarded as the autophagic activity [78]. We found that Beclin-1 and LC3-II/LC3-I were increased while mTOR and p-mTOR were decreased in ovalbumin-induced asthma model rats. EEL, Bud, and Bud&EEL could reduce Beclin-1 and LC3-II/LC3-I but promote mTOR and p-mTOR. In addition, the effect of Bud&EEL on regulating autophagy was better than that of single-use Bud. Our study indicates that Bud&EEL has synergistic effect on inhibiting autophagy induced in asthma rats, suggesting that inhibiting autophagy might be a possible mechanism by which the coadministration of Bud and EEL improves airway remodeling.

**Figure 8:**
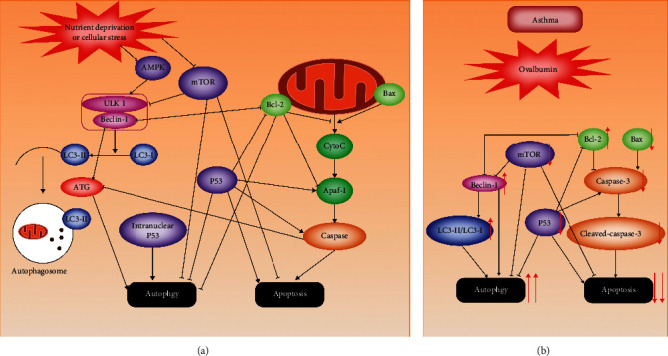
The crosstalk between autophagy and apoptosis. (a) In physiological situations, the activities of autophagy and apoptosis are influenced by many molecular signaling pathways, and some protein can regulate both autophagy and apoptosis. (b) In ovalbumin-induced asthmatic rats, the activity of autophagy is increased whereas apoptosis is reduced through detecting the expressions of autophagy-related proteins and apoptosis-related proteins.

As shown in Figure 8, autophagy has many crosstalks with apoptosis due to the interactions of autophagy- and apoptosis-related proteins [79], including the following: (i) the interaction between Bcl-2 and Beclin-1 inhibits autophagy. (ii) P53 exerts different effects on autophagy, according to the location of P53 in cell—nuclear p53 promotes it, whereas cytoplasmic p53 prevents it. P53 promotes apoptosis by increasing the expression of proapoptotic proteins such as Bax, Apaf-1, and Caspase-6 which mediates intrinsic and extrinsic apoptosis pathway. (iii) mTOR acts as an inhibitor on both autophagy and apoptosis. (iv) Activated caspases can inhibit autophagy by splitting autophagy-associated proteins, such as Beclin-1, ATG5, and p62 [80, 81]. In the present study, we observed that the mRNA and protein expressions of Bcl-2, Bax, Caspase-3, P53, Beclin-1, and mTOR make crosstalk between apoptosis and autophagy clear in asthma rats. Our study shows that apoptotic activity is decreased whereas autophagic activity is increased, and the interaction between them exists in ovalbumin-induced asthma rats. It confirms that disordered apoptosis and autophagy are asthmatic pathogenesis, providing researchers with ideas on the development of new drugs for treating asthma. However, the complicated mechanism of their interaction is not explained clearly, warranting further investigation of that.

## 5. Conclusions

In summary, coadministration of Bud and EEL had superior effects on attenuating airway remodeling; promoting apoptosis; inhibiting autophagy; reducing the expressions of Bcl-2 and Beclin-1; increasing the expressions of Bax, Caspase-3, cleaved Caspase-3, P53, mTOR, and p-mTOR; and regulating Bcl-2/Bax and LC3-II/LC3-I compared with single-Bud treatment. Our study shows that restoring apoptosis and autophagy—promoting apoptosis and inhibiting autophagy—might be possible mechanisms of combined extracts of EF and LLF with budesonide on improving airway remodeling in ovalbumin-induced asthma rats, providing some ideas for treating asthma in the future. The molecular mechanisms of interplay between autophagy and apoptosis in asthma need further study.

## Figures and Tables

**Table 1:** Antibodies used for IF analysis.

Antibody	Description	Concentration	Manufacturer
*α*-SMA	Mouse	1 : 200	Bioss, Beijing, China
Ki-67	Rabbit	1 : 250	Abcam, Cambridge, UK
Bcl-2	Mouse	1 : 200	Santa Cruz Biotechnology, Santa Cruz, USA
Bax	Rabbit	1 : 100	Abcam, Cambridge, UK
Caspase-3	Rabbit	1 : 200	Abcam, Cambridge, UK
P53	Mouse	1 : 200	Santa Cruz Biotechnology, Santa Cruz, USA
mTOR	Rabbit	1 : 100	Abcam, Cambridge, UK
Beclin-1	Rabbit	1 : 200	Abcam, Cambridge, UK

**Table 2:** Antibodies used for WB analysis.

Antibody	Description	Concentration	Manufacturer
Bcl-2	Mouse	1 : 700	Santa Cruz Biotechnology, Santa Cruz, USA
Bax	Rabbit	1 : 1000	Abcam, Cambridge, UK
Caspase-3	Rabbit	1 : 700	Abcam, Cambridge, UK
Cleaved Caspase-3	Rabbit	1 : 1000	Abcam, Cambridge, UK
Beclin-1	Rabbit	1 : 1000	Abcam, Cambridge, UK
LC3A/B	Rabbit	1 : 1000	Abcam, Cambridge, UK
P53	Mouse	1 : 700	Santa Cruz Biotechnology, Santa Cruz, USA
mTOR	Rabbit	1 : 2000	Abcam, Cambridge, UK
p-mTOR	Rabbit	1 : 1000	Cell Signaling Technology, Boston, USA

**Table 3:** Primers used for qPCR analysis.

Primer	Forward primer	Reverse primer
Bax	CCAAGAAGCTGAGCGAGTGT	TCACGGAGGAAGTCCAGTGT
Bcl-2	AGCATGCGACCTCTGTTTGA	TCACTTGTGGCCCAGGTATG
Caspase-3	TCTACCGCACCCGGTTACTA	CGTACAGTTTCAGCATGGCG
P53	TCGGCTCCGACTATACCACT	GTCCCGTCCCAGAAGATTCC
mTOR	GCTTATCAAGCAAGCGACATCTCA	TCCACTGGAAGCACAGACCAAG
Beclin-1	TTGGCCAATAAGATGGGTCTGAA	TGTCAGGGACTCCAGATACGAGTG
*β*-actin	AGCCATGTACGTAGCCATCC	ACCCTCATAGATGGGCACAG

## Data Availability

The data used to support the findings of this study are available from the corresponding author upon request.

## Abbreviations

BALF: Bronchoalveolar lavage fluid
Bud: Budesonide
Bud&EEL: Coadministration of budesonide with the extracts of *Epimedii Folium* and *Ligustri Lucidi Fructus*
Caspases: Cysteine aspartic proteinases
EF: *Epimedii Folium*
EEL: The extracts of *Epimedii Folium* and *Ligustri Lucidi Fructus*
GCs: Glucocorticoids
H&E: Hematoxylin and eosin
HPA: Hypothalamic-pituitary-adrenal
IL: Interleukin
IF: Immunofluorescence
IOD: Integral optical density
i.p.: Intraperitoneal injection
i.g.: Gastric perfusion
Inhal.: Aerosol inhalation
LLF: Ligustri Lucidi Fructus
PAS: Periodic acid–Schiff
Pbm: Perimeter of basement membrane
p-mTOR: Phospho-mTOR
qPCR: Quantitative real-time PCR
SD: Standard deviation
s.c.: Subcutaneous injection
TUNEL: TdT-dUTP nick-end labeling
TCM: Traditional Chinese Medicine
Wat: Thickness of airway wall
Wam: Thickness of airway smooth muscle
WB: Western blotting
*α*-SMA: Alpha smooth muscle actin.
